# Association of sodium voltage-gated channel genes polymorphisms with epilepsy risk and prognosis in the Saudi population

**DOI:** 10.1080/07853890.2022.2096257

**Published:** 2022-07-08

**Authors:** Mansour A. Alghamdi, Laith N. AL-Eitan, Ashwag Asiri, Doaa M. Rababa’h, Sultan A. Alqahtani, Mohammed S. Aldarami, Manar A. Alsaeedi, Raghad S. Almuidh, Abdulbari A. Alzahrani, Ahmad H. Sakah, Eman Mohamad El Nashar, Mansour Y. Otaif, Nawal F. Abdel Ghaffar

**Affiliations:** a Department of Anatomy, College of Medicine, King Khalid University, Abha, Saudi Arabia; b Genomics and Personalized Medicine Unit, College of Medicine, King Khalid University, Abha, Saudi Arabia; c Department of Applied Biological Sciences, Jordan University of Science and Technology, Irbid, Jordan; d Department of Biotechnology and Genetic Engineering, Jordan University of Science and Technology, Irbid, Jordan; e Department of Child Health, College of Medicine, King Khalid University, Abha, Saudi Arabia; f Neurology Department, Neuroscience Centre, King Faisal Specialist Hospital & Research Center, Riyadh, Saudi Arabia; g College of Medicine, Alfaisal University, Riyadh, Saudi Arabia; h Department of Histology and Cell Biology, Faculty of Medicine, Benha University, Benha, Egypt; i Department of Pediatric, Neurology section, Abha Maternity and Childern Hospital, Abha, Saudi Arabia; j Neurology Department, Kasr Al Ainy Hospital, Faculty of Medicine, Cairo University, Giza, Egypt; k Neurology Department, Aseer Central Hospital, Abha, Saudi Arabia

**Keywords:** Epilepsy, genetic variant, prognosis, SCN

## Abstract

**Background:**

Epilepsy is a heterogeneous complex condition that involve the human brain. Genetic predisposition to epilepsy is a fundamental factor of the disorder aetiology. The sodium voltage-gated channel (SCN) genes variants are critical biomarker for the epilepsy development and progression. In this study, we aimed to investigate the association of several SNCs genetic polymorphisms with epilepsy risk and their intrudance of the disease prognosis.

**Methods:**

Blood samples were withdrawn from 296 Epilepsy patients in addition to 293 healthy matched participants prior to DNA extraction. PCR-sequencing was used for genotyping analysis. Genotyping outputs were then statistically analysed for genotype/phenotype evaluation.

**Results:**

Within SCN1A gene we found that the rs6432861 (*p* = 0.014) was in correlation with the risk of epilepsy. In addition, both rs4667485 and rs1469649 of SCN2A gene were significantly correlated to epilepsy risk for both allelic (4e-4 and 1e-3) and genotypic (1e-3 and 5e-3). Moreover, the haplotype analysis showed that the GATGCTCGGTTTCGCTACGCA haplotype of SCN2A gene was significantly related to epilepsy increased risk, *p* = 6e-3, OR (CI) = 2.02 (1.23–3.31). In relevant to our finding, many of the investigated SCNs variants in the current study were related to several clinical features of epilepsy.

**Conclusion:**

In light of our results, we infer that SCN genes polymorphisms are strong candidates for epilepsy development and progression. Furthermore, these variant are essential for the disorder prognosis and medications outcomes.

**Key Messages**
- Genetic polymorphisms of sodium channels SCN1A, SCN2A and SCN3A were found to be associated with the risk of epilepsy.
- SCN1B polymorphisms were found to be correlated to epilepsy reduced risk.
- SCNs variants are involved in the epilepsy prognosis and response to treatment.

## Introduction

Epilepsy is a major neurological disorder in which heterogeneous conditions impact the human’s health [1]. The main characterization of epilepsy is recurrent seizures that happen due to a sudden abnormal electrical activity in the central nervous system [2]. There are several factors that contribute to the development of epilepsy including environmental and genetic factors . Precisely, epilepsy onset is attributed to the interaction between both the environment and the genetic factors. Besides, it considered as a complex disorder. Two major types of epilepsy were identified according to seizures; generalized seizures that produced through the entire brain and focal seizures that occur in a local part of the brain [3].

Molecular studies have reported that genetic markers of crucial genes may influence epilepsy risk. These genes are involved in different neurological pathways such as voltage-gated sodium [4], potassium, calcium, chloride channels, acetylcholine and γ-amino butyric acid [5,6]. The sodium voltage-gated channel (SCN) proteins play a fundamental roles in the generation of action potential and found in neuronal, endocrine cells and consisted of either alpha or beta subunit [7]. SCN mediates the influx of sodium ions that are important for neurons to initiate the action potential. Therefore, mutations within SCN genes may impact epilepsy development and progression [8].

SCN1A has been intensively studied regarding the risk of epilepsy. These genes are located on chromosome 2 and consist of 26 exons that spans over 100 kb of genomic DNA [9]. This gene was implicated in Genetic Epilepsy with Febrile Seizures Plus (GEFS+) and Familial Febrile Seizures (FFS). GEFS + is an epilepsy syndrome that is detected within families, while FFS common in children from six months to five years [1]. In addition, SCN1A was involved in Dravet syndrome (DS) which is severe genetic epileptic encephalopathy with an increased risk of sudden and unexpected death of someone with epilepsy [10]. Regarding genetic vulnerability to epilepsy, different types of mutation have been reported in *SCN1A* including frameshift, nonsense and splice site mutations. Moreover, about 30 missense mutants that alter the SCN1A activity have been exposed. These missense mutations may also obliterate channel function by manipulating the properties of the channel, trafficking or subcellular localization [11]. The association between several single variants with epilepsy risk and drug response has been elaborated. In Italy, three variants of SCN1A: rs6730344, rs6732655 and rs10167228 were reported to impact the drug resistance to epilepsy [12]. On the other hand, a study in Hong Kong and Malaysia, Patino et al. [13] and Ramadan et al. [14] investigated the influence of common polymorphisms within SCN1AA (rs3812718 and rs2298771), SCN2A (rs17183814) on drug response to epilepsy and found no correlation between these variants and anti-epileptic drug (AEDs) [15]. Recently, SCN2A mutations have been implicated with epilepsy of infancy with migrating focal seizures (EIMFS) syndrome [16,17], benign familial neonatal infantile seizures (BFNIS) and early infantile epileptic encephalo-pathy (EIEE) [18]. Several variants of SCN2A gene (rs10197716, rs2119068, rs2119067, rs353116, rs353112 and rs6740895) have been proposed to be associated with Valproic Acid (VPA) responses in Chinese Han epileptic patients [18].

The available data that describe the association between SCN3A variants and epilepsy are limited and few were correlated to focal epilepsy [19]. rs1057518801 and rs1057520753 of SCN3A manifest a gain of function in sodium channels that results in more hyperpolarized potentials and had been linked to early infantile epileptic encephalopathy [20]. SCN1B and SCN2B gene have been associated with GEFS [21,22] and dravet syndrome [13,23]. rs786205830 is a loss of function mutation in the SCN1B gene had been linked to early infantile developmental and epileptic encephalopathy (Aeby et al. 2019). SCN8A has been reported to influence epilepsy at four months of early life and can affect the development. This gene may also enhance several epilepsy syndromes such as Lennox-Gastaut syndrome, West syndrome and epileptic encephalopathies (e.g. Dravet syndrome) [16]. Moreover, SCN8A also related to EIEE and BFIS (Makoff et al. 2009).

Hence, the aforementioned genes of sodium voltage-gated channels and their polymorphisms were represented as risk factor for epilepsy; this study aims to investigate the association between several genetic variants of SCN genes and epilepsy in Saudi population. In addition, in this study, genotype–phenotype analyses were conducted to reveal the relationship between the genetic variants of SCNs and several clinical and prognostic factors of epilepsy patients.

## Materials and methods

### Study subjects and design

This study involved 296 individuals who were diagnosed with Epilepsy in addition to 293 healthy matched participants. The study cohorts were Arabs from Saudi Arabia. Epileptic patients were recruited from neurology clinics at Asir Central Hospital, Abha Maternity and Childern Hospital, Khamis Mushait General Hospital and Primary Health Care Centre in Almansak in addition to their medical records. Written informed consent was obtained from all study subjects, and ethical approval to carry out this study was obtained from the Research Ethics Committee at King Khalid University (Reference No. ECM#2019-38).

### DNA extraction and genotyping

Genomic DNA was purified from peripheral blood sample using the Wizard Genomic DNA Purification kit (Promega Corporation, Madison, WI, USA). The quality and quantity of the purified DNA were obtained using agarose gel electrophoresis and the Nano-Drop ND-1000 UV-Vis Spectrophotometer (BioDrop, UK), respectively. DNA samples were then diluted with nuclease-free water to achieve a final concentration of 20 ng/μl and a final volume ranging between 50 and 500 μl. Afterwards, samples were shipped on ice to Gehrmann Laboratories, The University of Queensland, QLD 4072 for custom genotyping on the Sequenom MassARRAY® system (iPLEX GOLD) (Sequenom, USA).

### Statistical analyses

Both the Hardy–Weinberg equilibrium (p^2^ + 2pq + q^2^ = 1) (http://www.oege.org/software/hwe-mr-calc.html) was applied to test the SNPs. snpSTAT, © 2006 Institut Català d'Oncologia software was used to assess the genotypic and allelic frequencies in addition to the genetic association. Data description and phenotype-genotype analyses were conducted using the JASP 0.16 software. For the present study, statistical significance for single testing was set at *p* value <.05. Bonferroni correction was performed to the multiple testing using the sets of the significance cut-off at (α/n) where α = 0.05 and n number of tests, and to sustain the overall *p*-value at significance level of 0.025 or less.

## Results

### Patient’s characteristics

Table 1 displays several clinical features of epilepsy in addition to the general parameters. The study cohort consisted of males and females at similar ratio. The mean age of the participants was 9.7 ± 7.986 and ranged from few months to 35 years old. Within patients, the average of the body mass index, BMI indicated a healthy weight (23.2 ± 7.789), as the table shows. The majority of the participants were diagnosed with generalized epilepsy. About 55% of the patients had relapse, while 79% had periodic epilepsy. The response to the first medication for the epileptic patients in the current study was good that accounted for 64%, while most of the cohort did not take any ADE medications. In this study, we estimated that 33.9% of patients recorded with family history of epilepsy; meanwhile 11.9% had a history of febrile seizure. Patients were classified also according to seizure type, and the table depicts that 94.6% of them had motor movement seizure; 52% of patients were observed with seizure free immediately after medication compared with 18% who needed six months to reach seizure-free status.

**Table 1.** General and clinical characteristic of the epilepsy cohort.

Parameter	Mean/frequency	
Age at diagnosis	9.7 ± 7.986	
BMI	23.2 ± 7.789	
Duration of first seizure (sec)	8.703 ± 14.032	
Gender	Male	51%
	Female	49%
Time to remission	No	28.5%
	Yes (seizure free immediately)	52.3%
	Yes (seizure free within 6 months)	18.7%
Epilepsy classification	Focal (partial) Onset	16%
	Generalized Onset	76%
	Combined generalized and focal Onset	8%
Seizure classification	Motor	94.6%
	Non-motor	5.4%
History of febrile seizure	Yes	11.9%
	No	88.1%
Family history of epilepsy	Yes	33.9%
	No	66.1%
AED Drug	Yes	6%
	No	94%
First drug responsiveness	Yes	64%
	No	36%
Relapse	Yes	45%
	No	55%
Periodic epilepsy	Yes	21%
	No	79%

### Candidate SNPs

Table 2 describes the information regarding the genetic variants of SCN superfamily among epilepsy cases and healthy controls. Minor alleles and their frequencies, and *p* value of HWE in addition to the chromosomal position of the SNPs are listed in the table. The non-polymorphic and SNPs that did not fulfil the HWE were excluded from this study.

**Table 2.** Description of SCN genes variants among epilepsy cases and healthy controls.

Gene	SNP ID	SNP position^a^	Variant Consequence	Cases (*n* = 296)			Controls (*n* = 293)	
				MA	MAF	HWE *p* value	MAF	HWE *p* value
SCN1A	rs3812718	2:166053034	Intron	T	0.46	0.73	0.40	0.05
	rs2298771	2:166036278	Missense	C	0.46	0.41	0.49	0.91
	rs10167228	2:166042592	Intron	T	0.46	0.41	0.49	0.91
	rs146733308	2:165991324	Missense	T	0.00	N/A	0.00	N/A
	rs35735053	2:165991411	Missense	G	0.00	N/A	0.00	N/A
	rs3749029	2:165991504	Missense	T	0.00	N/A	0.00	N/A
	rs6432858	2:166033433	Intron	C	0.46	0.41	0.49	1.00
	rs10194956	2:166046065	Intron	G	0.46	0.72	0.40	0.06
	rs146878122	2:166009822	Missense	A	0.00	0.10	0.00	N/A
	rs121918817	2:166045080	Missense	T	0.00	N/A	0.00	N/A
	rs121918624	2:166052882	Stop Gained	A	0.00	0.10	0.00	0.10
	rs2169312	2:166071386	Intron	C	0.17	0.68	0.18	0.69
	rs1461197	2:166067962	Intron	A	0.33	0.11	0.36	0.08
	rs13405797	2:166062652	Intron	A	0.17	0.84	0.17	0.53
	rs4667866	2:166059523	Intron	G	0.05	0.59	0.06	0.61
	rs10188577	2:166059387	Intron	C	0.28	0.001	0.23	0.87
	rs1020853	2:166023272	Intron	T	0.22	1.00	0.22	0.86
	rs7587026	2:166122240	Intron	A	0.24	0.01	0.19	1.0
	rs1381105	2:166068661	Intron	G	0.24	0.43	0.25	0.54
	rs2162600	2:166069891	Intron	C	0.18	0.55	0.18	0.69
	rs10182473	2:166017213	Intron	C	0.22	1.00	0.22	0.87
	rs16851381	2:166056929	Intron	G	0.05	1.00	0.06	0.61
	rs4667869	2:166066849	Intron	G	0.18	0.55	0.17	0.55
	rs11692675	2:166069918	Intron	C	0.29	1e-3	0.24	0.53
	rs10497275	2:165990220	Intron	G	0.05	0.59	0.06	0.61
	rs7577411	2:165989284	Non Coding Transcript	A	0.02	1.00	0.03	1e-3
	rs10930195	2:166045896	Intron	A	0.46	0.72	0.40	0.04
	rs6432861	2:166046718	Intron	T	0.45	0.051	0.40	0.14
	rs7580482	2:166046935	Synonymous	T	0.48	0.73	0.51	0.48
	rs13383628	2:166047150	Intron	T	0.46	0.64	0.4	0.051
	rs1841546	2:166052594	Intron	T	0.43	0.53	0.4	0.03
	rs6722462	2:166075016	Intron	C	0.33	0.09	0.36	0.10
	rs6432860	2:166041354	Synonymous	A	0.46	0.35	0.49	1.00
	rs994399	2:166048970	Intron	G	0.48	0.73	0.51	0.41
	rs1542484	2:166048865	Intron	A	0.46	0.72	0.4	0.05
	rs1461193	2:166047836	Intron	G	0.46	0.41	0.49	0.91
	rs11690959	2:166047515	Intron	A	0.45	0.73	0.40	0.07
	rs7601520	2:166036571	Intron	G	0.45	0.40	0.49	0.81
SCN2A	rs12467383	2:165369259	Intron	A	0.06	1.00	0.07	1.00
	rs2304016	2:165311993	Intron	G	0.00	N/A	0.00	N/A
	rs17183814	2:165295879	Missense	A	0.04	1e-3	0.04	5e-4
	rs1965757	2:165308377	Intron	G	0.29	0.16	0.32	0.68
	rs353139	2:165289717	Intron	C	0.36	0.30	0.38	0.90
	rs7581811	2:165300655	Intron	C	0.36	0.8	0.39	0.90
	rs2116658	2:165310162	Intron	T	0.38	0.08	0.41	0.33
	rs12993173	2:165326704	Intron	G	0.37	0.45	0.40	1.00
	rs4667485	2:165363235	Intron	C	0.28	0.89	0.20	0.58
	rs1469649	2:165370597	Intron	G	0.29	0.78	0.21	1.00
	rs3816002	2:165342576	Intron	G	0.00	N/A	0.00	N/A
	rs1579865	2:165335204	Intron	A	0.37	0.17	0.42	0.54
	rs935403	2:165328783	Intron	C	0.34	0.24	0.38	0.80
	rs7592445	2:165303176	Intron	C	0.29	0.26	0.31	0.78
	rs2119067	2:165270773	Intron	C	0.18	1.00	0.16	0.66
	rs2119068	2:165270664	Intron	C	0.42	0.90	0.43	1.00
	rs10197716	2:165268464	Intron	G	0.27	0.30	0.27	0.12
	rs353112	2:165280573	Intron	C	0.45	0.10	0.44	0.40
	rs6740895	2:165283510	Intron	C	0.23	0.11	0.26	0.37
	rs3943809	2:165344371	Intron	G	0.06	1.00	0.07	1.00
	rs16850331	2:165292743	Intron	T	0.07	0.63	0.08	1.00
	rs12614399	2:165293790	Intron	C	0.07	0.63	0.08	1.00
	rs10182570	2:165253124	Intron	C	0.26	0.45	0.27	0.18
	rs1446579	2:165267091	Intron	G	0.46	0.41	0.47	0.56
*SCN3A*	rs7598098	2:165109768	Intron	C	0.22	0.49	0.26	0.36
	rs11903851	2:165134943	Intron	T	0.26	<1e-4	0.25	1.00
	rs1439807	2:165143358	Intron	G	0.28	<1e-4	0.26	0.88
	rs12615864	2:165175282	Intron	A	0.07	0.14	0.09	0.72
	rs16850186	2:165167410	Intron	A	0.10	0.33	0.14	0.22
	rs1439806	2:165135256	Intron	A	0.26	0.35	0.23	0.17
	rs7596422	2:165151706	Intron	G	0.50	0.56	0.48	0.82
*SCN1B*	rs3746255	19:35033642	Synonymous	T	0.00	N/A	0.00	N/A
	rs745394799	19:35034182	Intron	T	0.00	N/A	0.00	N/A
	rs2305748	19:35039234	Missense	T	0.00	N/A	0.00	N/A
	rs72550243	19:35039332	Intron	T	0.00	1.00	0.00	1.00
	rs55742440	19:35033920	Missense	T	0.45	0.03	0.43	0.72
	rs67701503	19:35034035	Missense	A	0.19	0.01	0.16	1.00
	rs67486287	19:35034040	Missense	C	0.18	0.02	0.15	1.00
*SCN2B*	rs8192613	11:118167098	Intron	T	0.38	0.81	0.40	0.27
	rs8192614	11:118166849	3 Prime UTR	A	0.13	1.00	0.13	0.11
*SCN3B*	rs3851100	11:123653202	Intron	C	0.07	0.18	0.07	0.64
	rs1783901	11:123642798	Intron	T	0.28	0.19	0.24	0.33
*SCN8A*	rs303777	12:51754412	Intron	A	0.27	0.1	0.25	0.76
	rs11169883	12:51597548	Intron	T	0.41	0.06	0.46	0.24
	rs303778	12:51750944	Intron	G	0.15	1.00	0.13	1.00

^a^Chromosome positions are based on NCBI Human Genome Assembly Build.

MA: minor allele; MAF: minor allele frequency; HWE: Hardy—Weinberg equilibrium.

#### Genetic association between SCN genetic variants and epilepsy

**Figure 1.**
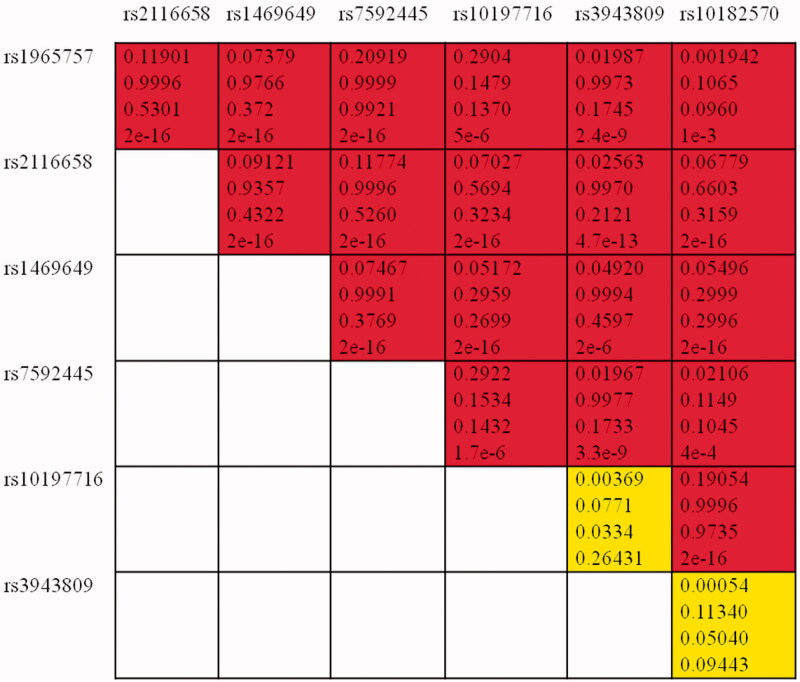
Linkage disequilibrium (LD) plots of variants within SCN2A. Values in the boxes from top to bottom are D, D′, r and *p* value. Boxes without numbers have D′= 1. Red boxes indicate a significant, whereas yellow boxes indicate insignificant variants.

The correlation between the investigated SNPs and the risk of epilepsy is illustrated in Table 3. Both allelic and genotypic frequencies among patients and controls were calculated. The significant of the association was estimated depending on the *p* value. As the table shows, there was no statistically significant difference in the genotypic distribution among cases and controls of rs3812718 (SCN1A) (*p* = .039). However, the distribution of the variant allele among patients (13%) was lower than it among controls (21%). This indicates that the TT genotype may be a protective factor against epilepsy and may decrease the risk of the disease. Our results also illustrated that the variant genotypes of rs13383628 (TT) may influence the risk of epilepsy (*p* = .014). Both rs4667485 and rs1469649 of SCN2A gene were significantly correlated to epilepsy risk for both allelic (4e-4 and 1e-3) and genotypic (1e-3 and 5e-3) association according to Table 3. We propose that the C/CC of rs4667485 as well as the G/GG of rs1469649 may act as increased risk factor for epilepsy development and progression. This study findings also revealed that the minor allele A of the rs16850186 in SCN3A was higher within controls than within patients but no compelling association was detected (*p* = 0.03). Finally, the haplotype analysis revealed that the GATGCTCGGTTTCGCTACGCA haplotype of SCN2A polymorphisms (see Table 3) was significantly related to epilepsy increased risk, *p* = 6e-3, OR (CI) = 2.02 (1.23–3.31). Linkage disequilibrium analysis was performed as Figure 1 shows, and seven SNPs were found as casual variants within the significant SCN2A haplotype.

**Table 3.** Genetic association between the SCN polymorphisms and epilepsy.

Gene	SNP ID	Allelic and Genotypic Frequencies in Cases and Controls			
		Allele/ Genotype	Cases (*n* = 296)	Controls (*n* = 293)	*p* value*
SCN1A	rs3812718	C	322 (54%)	349 (60%)	.06
		T	270 (46%)	235 (40%)	
		C/C	96 (32.9%)	89 (30.1%)	.039
		C/T	157 (53.8%)	144 (48.6%)	
		T/T	39 (13.4%)	63 (21.3%)	
	rs2298771	T	319 (54%)	297 (51%)	.27
		C	271 (46%)	287 (49%)	
		T/T	90 (30.5%)	76 (26%)	.48
		C/T	139 (47.1%)	145 (49.7%)	
		C/C	66 (22.4%)	71 (24.3%)	
	rs10167228	A	319 (54%)	294 (51%)	.25
		T	2714 6 (%)	286 (49%)	
		A/A	90 (30.5%)	75 (25.9%)	.45
		A/T	139 (47.1%)	144 (49.7%)	
		T/T	66 (22.4%)	71 (24.5%)	
	rs6432858	T	320 (54%)	295 (51%)	.25
		C	272 (46%)	287 (49%)	
		T/T	90 (30.4%)	75 (25.8%)	.45
		T/C	140 (47.3%)	145 (49.8%)	
		C/C	66 (22.3%)	71 (24.4%)	
	rs10194956	G	318 (54%)	334 (60%)	.06
		A	266 (46%	224 (40%)	
		G/G	88 (30.1%)	92 (33%)	.041
		A/G	142 (48.6%)	150 (53.8%)	
		A/A	62 (21.2%)	37 (13.3%)	
	rs2169312	T	473 (83%)	478 (82%	.70
		C	97 (17%)	104 (18%)	
		T/T	197 (69.1%)	195 (67%)	.78
		T/C	79 (27.7%)	88 (30.2%)	
		C/C	9 (3.2%)	8 (2.8%)	
	rs1461197	G	390 (67%)	372 (64%)	.27
		A	192 (33%)	210 (36%)	
		G/G	137 (47.1%)	126 (43.3%)	.57
		G/A	116 (39.9%)	120 (41.2%)	
		A/A	38 (13.1%)	45 (15.5%)	
	rs13405797	G	490 (83%)	486 (83%)	.84
		A	102 (17%)	98 (17%)	
		G/G	202 (68.2%)	200 (68.5%)	.87
		G/A	86 (29.1%)	86 (29.4%)	
		A/A	8 (2.7%)	6 (2%)	
	rs4667866	A	560 (95%)	550 (94%)	.76
		G	32 (0.05%)	1834 (0.06%)	
		A/A	265 (89.5%)	258 (88.4%)	.43
		A/G	30 (10.1%)	34 (11.6%)	
		G/G	1 (0.3%)	0 (0%)	
	rs1020853	G	459 (78%)	455 (78%)	.95
		T	129 (22%)	129 (22%)	
		G/G	179 (60.9%)	178 (61%)	.98
		G/T	101 (34.4%)	99 (33.9%)	
		T/T	14 (4.8%)	15 (5.1%)	
	rs1381105	T	448 (76%)	436 (75%)	.54
		G	140 (24%)	148 (25%)	
		T/T	173 (58.8%)	165 (56.5%)	.84
		G/T	102 (34.7%)	106 (36.3%)	
		G/G	19 (6.5%)	21 (7.2%)	
	rs2162600	T	487 (82%)	479 (82%)	.91
		C	105 (18%)	105 (18%)	
		T/T	202 (68.2%)	195 (66.8%)	.68
		T/C	83 (28%)	89 (30.5%)	
		C/C	11 (3.7%)	8 (2.7%)	
	rs10182473	T	457 (78%)	451 (78%)	N/A
		C	131 (22%)	129 (22%)	
		T/T	177 (60.2%)	176 (60.7%)	.96
		C/T	103 (35%)	99 (34.1%)	
		C/C	14 (4.8%)	15 (5.2%)	
	rs16851381	A	560 (95%)	549 (94%)	.84
		G	32 (0.05%)	33 (6%)	
		A/A	265 (89.5%)	258 (88.7%)	.45
		A/G	30 (10.1%)	33 (11.3%)	
		G/G	1 (0.3%)	0 (0%)	
	rs4667869	C	483 (82%)	481 (83%)	.82
		G	105 (18%)	101 (17%)	
		C/C	200 (68%)	197 (67.7%)	.61
		G/C	83 (28.2%)	87 (29.9%)	
		G/G	11 (3.7%)	7 (2.4%)	
	rs10497275	A	560 (95%)	551 (94%)	.85
		G	32 (0.05%)	33 (.06%)	
		A/A	265 (89.5%)	259 (88.7%)	.46
		G/A	30 (10.1%)	33 (11.3%)	
		G/G	1 (0.3%)	0 (0%)	
	rs6432861	C	303 (55%)	343 (60%)	.11
		T	243 (45%)	227 (40%)	
		C/C	92 (33.7%)	97 (34%)	.014
		T/C	119 (43.6%)	149 (52.3%)	
		T/T	62 (22.7%)	39 (13.7%)	
	rs7580482	C	306 (52%)	284 (49%)	.32
		T	284 (48%)	296 (51%)	
		C/C	81 (27.5%)	66 (22.8%)	.42
		C/T	144 (48.8%)	152 (52.4%)	
		T/T	70 (23.7%)	72 (24.8%)	
	rs13383628	T	320 (54%)	347 (60%)	.07
		C	268 (46%)	235 (40%)	
		T/T	89 (30.3%)	95 (32.6%)	.036
		T/C	142 (48.3%)	157 (54%)	
		C/C	63 (21.4%)	39 (13.4%)	
	rs6722462	T	394 (67%)	372 (64%)	.30
		C	198 (33%)	212 (36%)	
		T/T	138 (46.6%)	125 (42.8%)	.61
		T/C	118 (39.9%)	122 (41.8%)	
		C/C	40 (13.5%)	45 (15.4%)	
	rs6432860	G	319 (54%)	295 (51%)	.27
		A	273 (46%)	287 (49%)	
		G/G	90 (30.4%)	75 (25.8%)	.46
		G/A	139 (47%)	145 (49.8%)	
		A/A	67 (22.6%)	71 (24.4%)	
	rs994399	A	306 (52%)	285 (49%)	.32
		G	284 (48%)	297 (51%)	
		A/A	81 (27.5%)	66 (22.7%)	.41
		A/G	144 (48.8%)	153 (52.6%)	
		G/G	70 (23.7%)	72 (24.7%)	
	rs1542484	A	315 (54%)	348 (60%)	.05
		G	265 (46%)	232 (40%)	
		A/A	87 (30%)	96 (33.1%)	.03
		G/A	141 (48.6%)	156 (53.8%)	
		G/G	62 (21.4%)	38 (13.1%)	
	rs1461193	A	320 (54%)	297 (51%)	.27
		G	272 (46%)	287 (49)	
		A/A	90 (30.4%)	76 (26%)	.49
		G/A	140 (47.3%)	145 (49.7%)	
		G/G	66 (22.3%)	71 (24.3%)	
	rs11690959	G	321 (55%)	345 (60%)	.08
		A	267 (45%)	233 (40%	
		G/G	89 (30.3%)	95 (32.9%)	.05
		A/G	143 (48.6%)	155 (53.6%)	
		A/A	62 (21.1%)	39 (13.5%)	
	rs7601520	A	310 (55%)	291 (51%)	.19
		G	258 (45%)	283 (49%)	
		A/A	88 (31%)	75 (26.1%)	.40
		G/A	134 (47.2%)	141 (49.1%)	
		G/G	62 (21.8%)	71 (24.7%)	
SCN2A	rs12467383	G	554 (94%)	544 (93%)	.60
		A	36 (0.06%)	40 ( (0.07%)	
		G/G	260 (88.1%)	253 (86.6%)	.86
		G/A	34 (11.5%)	38 (13%)	
		A/A	1 (0.3%)	1 (0.3%)	
	rs1965757	A	419 (71%)	397 (68%)	.39
		G	173 (29%)	183 (32%)	
		A/A	143 (48.3%)	134 (46.2%)	.51
		G/A	133 (44.9%)	129 (44.5%)	
		G/G	20 (6.8%)	27 (9.3%)	
	rs353139	T	365 (64%)	356 (62%)	.31
		C	201 (36%)	222 (38%)	
		T/T	122 (43.1%)	110 (38.1%)	.46
		T/C	121 (42.8%)	136 (47.1%)	
		C/C	40 (14.1%)	43 (14.9%)	
	rs7581811	G	380 (64%)	352 (61%)	.22
		C	212 (36%)	228 (39%)	
		G/G	123 (41.5%)	106 (36.5%)	.44
		C/G	134 (45.3%)	140 (48.3%)	
		C/C	39 (13.2%)	44 (15.2%)	
	rs2116658	C	365 (62%)	344 (59%)	.24
		T	219 (38%)	238 (41%)	
		C/C	121 (41.4%)	106 (36.4%)	.46
		C/T	123 (42.1%)	132 (45.4%)	
		T/T	48 (16.4%)	53 (18.2%)	
	rs12993173	T	367 (63%)	349 (60%)	.28
		G	213 (37%)	231 (40%)	
		T/T	119 (41%)	105 (36.2%)	.49
		G/T	129 (44.5%)	139 (47.9%)	
		G/G	42 (14.5%)	46 (15.9%)	
	rs4667485	G	424 (72%)	470 (80%)	4e-4
		C	168 (28%)	114 (20%)	
		G/G	151 (51%)	187 (64%)	1e-3
		C/G	122 (41.2%)	96 (32.9%)	
		C/C	23 (7.8%)	9 (3.1%)	
	rs1469649	A	412 (71%)	461 (79%)	1e-3
		G	168 (29%)	121 (21%)	
		A/A	145 (50%)	182 (62.5%)	5e-3
		A/G	122 (42.1%)	97 (33.3%)	
		G/G	23 (7.9%)	12 (4.1%)	
	rs1579865	G	370 (63%)	330 (58%)	.08
		A	214 (37%)	236 (42%)	
		G/G	123 (42.1%)	99 (35%)	.2
		G/A	124 (42.5%)	132 (46.6%)	
		A/A	45 (15.4%)	52 (18.4%)	
	rs935403	T	392 (66%)	360 (62%)	.18
		C	200 (34%)	216 (38%)	
		T/T	125 (42.2%)	111 (38.5%)	.32
		T/C	142 (48%)	138 (47.9%)	
		C/C	29 (9.8%)	39 (13.5%)	
	rs7592445	T	419 (71%)	398 (69%)	.33
		C	169 (29%)	182 (31%)	
		T/T	145 (49.3%)	135 (46.5%)	.5
		C/T	129 (43.9%)	128 (44.1%)	
		C/C	20 (6.8%)	27 (9.3%)	
	rs2119067	T	487 (82%)	491 (84%)	.41
		C	105 (18%)	93 (16%)	
		T/T	200 (67.6%)	205 (70.2%)	.65
		C/T	87 (29.4%)	81 (27.7%)	
		C/C	9 (3%)	6 (2%)	
	rs2119068	G	345 (58%)	333 (57%)	.71
		C	245 (42%)	247 (43%)	
		G/G	100 (33.9%)	95 (32.8%)	.93
		C/G	145 (49.1%)	143 (49.3%)	
		C/C	50 (16.9%)	52 (17.9%)	
	rs10197716	A	420 (73%)	395 (73%)	.94
		G	158 (27%)	147 (27%)	
		A/A	156 (54%)	149 (55%)	.92
		G/A	108 (37.4%)	97 (35.8%)	
		G/G	25 (8.7%)	25 (9.2%)	
	rs353112	G	323 (55%)	325 (56%)	.57
		C	269 (45%)	253 (44%)	
		G/G	95 (32.1%)	95 (32.9%)	.75
		G/C	133 (44.9%)	135 (46.7%)	
		C/C	68 (23%)	59 (20.4%)	
	rs6740895	T	454 (77%)	431 (74%)	.25
		C	138 (23%)	153 (26%)	
		T/T	179 (60.5%)	162 (55.5%)	.47
		C/T	96 (32.4%)	107 (36.6%)	
		C/C	21 (7.1%)	23 (7.9%)	
	rs3943809	A	553 (94%)	544 (93%)	.69
		G	37 (0.06%)	40 (0.07%)	
		A/A	259 (87.8%)	253 (86.6%)	.91
		G/A	35 (11.9%)	38 (13%)	
		G/G	1 (0.3%)	1 (0.3%)	
	rs16850331	C	551 (93%)	539 (92%)	.47
		T	39 (0.07%)	45 (0.08%)	
		C/C	258 (87.5%)	248 (84.9%)	.51
		T/C	35 (11.9%)	43 (14.7%)	
		T/T	2 (0.7%)	1 (0.3%)	
	rs12614399	G	552 (93%)	538 (0.92%)	.46
		C	40 (0.07%)	46 (0.08%)	
		G/G	258 (87.2%)	247 (84.6%)	.51
		C/G	36 (12.2%)	44 (15.1%)	
		C/C	2 (0.7%)	1 (0.3%)	
	rs10182570	A	438 (74%)	427 (73%)	.73
		C	152 (26%)	155 (27%)	
		A/A	165 (55.9%)	161 (55.3%)	.88
		C/A	108 (36.6%)	105 (36.1%)	
		C/C	22 (7.5%)	25 (8.6%)	
	rs1446579	G	316 (54%)	301 (52%)	.45
		A	268 (46%)	279 (48%)	
		G/G	89 (30.5%)	75 (25.9%)	.41
		G/A	138 (47.3%)	151 (52.1%)	
		A/A	65 (22.3%)	64 (22.1%)	
SCN3A	rs7598098	G	447 (78%)	428 (74%)	.15
		C	127 (22%)	148 (26%)	
		G/G	176 (61.3%)	162 (56.2%)	.38
		C/G	95 (33.1%)	104 (36.1%)	
		C/C	16 (5.6%)	22 (7.6%)	
	rs12615864	G	544 (93%)	531 (91%)	.16
		A	40 (0.07%)	53 (0.09%)	
		G/G	255 (87.3%)	242 (82.9%)	.30
		A/G	34 (11.6%)	47 (16.1%)	
		A/A	3 (1%)	3 (1%)	
	rs16850186	G	535 (90%)	502 (86%)	.03
		A	57 (10%)	80 (14%)	
		G/G	243 (82.1%)	219 (75.3%)	.1
		A/G	49 (16.6%)	64 (22%)	
		A/A	4 (1.4%)	8 (2.8%)	
	rs1439806	G	422 (74%)	441 (77%)	.22
		A	146 (26%)	129 (23%)	
		G/G	160 (56.3%)	166 (58.2%)	.08
		G/A	102 (35.9%)	109 (38.2%)	
		A/A	22 (7.8%)	10 (3.5%)	
	rs7596422	A	296 (50%)	299 (52%)	.64
		G	294 (50%)	281 (48%)	
		A/A	77 (26.1%)	78 (26.9%)	.86
		G/A	142 (48.1%)	143 (49.3%)	
		G/G	76 (25.8%)	69 (23.8%)	
SCN1B	rs72550243	C	592 (100%)	581 (99%)	.08
		T	0 (0%)	3 (0.01%)	
		C/C	296 (100%)	289 (99%)	.04
		C/T	0 (0%)	3 (1%)	
SCN2B	rs8192613	G	366 (62%)	351 (60%)	.54
		T	226 (38%)	233 (40%)	
		G/G	114 (38.5%)	110 (37.7%)	.69
		G/T	138 (46.6%)	131 (44.9%)	
		T/T	44 (14.9%)	51 (17.5%)	
	rs8192614	G	516 (87%)	506 (87%)	.96
		A	76 (13%)	74 (13%)	
		G/G	225 (76%)	224 (77.2%)	.56
		G/A	66 (22.3%)	58 (20%)	
		A/A	5 (1.7%)	8 (2.8%)	
SCN3B	rs3851100	T	534 (93%)	541 (93%)	.87
		C	42 (0.07%)	41 (0.07%)	
		T/T	249 (86.5%)	252 (86.6%)	.9
		T/C	36 (12.5%)	37 (12.7%)	
		C/C	3 (1%)	2 (0.7%)	
	rs1783901	C	426 (72%)	442 (76%)	.12
		T	166 (28%)	140 (24%)	
		C/C	158 (53.4%)	171 (58.8%)	.32
		C/T	110 (37.2%)	100 (34.4%)	
		T/T	28 (9.5%)	20 (6.9%)	
SCN8A	rs303777	G	424 (73%)	433 (75%)	.42
		A	158 (27%)	145 (25%)	
		G/G	160 (55%)	163 (56.4%)	.48
		G/A	104 (35.7%)	107 (37%)	
		A/A	27 (9.3%)	19 (6.6%)	
	rs11169883	C	351 (59%)	312 (54%)	.05
		T	241 (41%)	270 (46%)	
		C/C	112 (37.8%)	89 (30.6%)	.15
		T/C	127 (42.9%)	134 (46%)	
		T/T	57 (19.3%)	68 (23.4%)	
	rs303778	A	503 (85%)	509 (87%)	.28
		G	89 (15%)	75 (13%)	
		A/A	213 (72%)	221 (75.7%)	.54
		A/G	77 (26%)	67 (22.9%)	
		G/G	6 (2%)	4 (1.4%)	

*p value <0.025 was considered as significant after performing Bonferroni correction.

#### Association of the clinical factors of epilepsy with SCN genes variants

Tables 4 and 5 describe the relationship between different features of epilepsy and the investigated variants. Within SCN2A gene, we found that the rs10194956 and rs4667869 were related to the duration of first seizure (*p* = .04 and .01, respectively). The rs303778 of SCN8A was correlated to vitamin B12 concentration (*p* = 2e-3) (Table 4).

**Table 4.** Regression analysis of SCN variants and epilepsy features.

Gene	SNP ID	Age at diagnosis	Duration of first seizure	BMI	VIT.D	VIT B12
SCN1A	rs3812718	0.428	0.193	0.261	0.899	0.532
	rs2298771	0.993	0.399	0.757	0.413	0.134
	rs10167228	0.640	0.094	0.425	0.153	0.495
	rs6432858	0.994	0.460	0.745	0.413	0.134
	rs10194956	0.149	0.043	0.323	0.065	0.807
	rs2169312	0.598	0.070	0.614	0.383	0.622
	rs1461197	0.891	0.510	0.378	0.552	0.125
	rs13405797	0.435	0.093	0.337	0.431	0.350
	rs4667866	0.071	0.997	0.897	0.082	0.310
	rs1020853	0.950	0.141	0.119	0.547	0.807
	rs1381105	0.503	0.153	0.289	0.242	0.580
	rs2162600	0.597	0.67	0.567	0.431	0.530
	rs10182473	0.611	0.264	0.168	0.179	0.580
	rs16851381	0.071	0.997	0.897	0.082	0.310
	rs4667869	0.812	0.010	0.239	0.434	0.350
	rs10497275	0.071	0.997	0.897	0.082	0.310
	rs6432861	0.425	0.147	0.713	0.702	0.645
	rs7580482	0.710	0.102	0.342	0.184	0.495
	rs13383628	0.146	0.057	0.254	0.087	0.807
	rs6722462	0.811	0.565	0.310	0.552	0.125
	rs6432860	0.926	0.486	0.894	0.413	0.134
	rs994399	0.125	0.722	0.335	0.184	0.495
	rs1542484	0.388	0.150	0.353	0.865	0.532
	rs1461193	0.652	0.117	0.146	0.153	0.495
	rs11690959	0.052	0.165	0.231	0.087	0.807
	rs7601520	0.433	0.071	0.522	0.259	0.495
SCN2A	rs12467383	0.544	0.254	0.888	0.913	0.968
	rs1965757	0.663	0.230	0.913	0.787	0.138
	rs353139	0.471	0.084	0.892	0.941	0.435
	rs7581811	0.809	0.810	0.583	0.959	0.766
	rs2116658	0.888	0.516	0.315	0.575	0.893
	rs12993173	0.826	0.798	0.848	0.748	0.766
	rs4667485	0.705	0.122	0.825	0.518	0.215
	rs1469649	0.452	0.693	0.869	0.858	0.339
	rs1579865	0.931	0.244	0.295	0.294	0.893
	rs935403	0.464	0.588	0.618	0.814	0.436
	rs7592445	0.591	0.226	0.892	0.787	0.138
	rs2119067	0.581	0.101	0.541	0.662	0.523
	rs2119068	0.388	0.632	0.630	0.243	0.881
	rs10197716	0.663	0.332	0.815	0.664	0.785
	rs353112	0.867	0.822	0.295	0.924	0.005
	rs6740895	0.973	0.136	0.373	0.411	0.865
	rs3943809	0.793	0.234	0.962	0.998	0.968
	rs16850331	0.652	0.251	0.909	0.922	0.968
	rs12614399	0.795	0.234	0.822	0.998	0.968
	rs10182570	0.405	0.598	0.536	0.429	0.793
	rs1446579	0.300	0.796	0.466	0.206	0.881
SCN3A	rs7598098	0.931	0.288	0.169	0.747	0.533
	rs12615864	0.315	0.844	0.176	0.220	N/A
	rs16850186	0.997	0.812	0.717	0.154	0.312
	rs1439806	0.271	0.754	0.984	0.834	0.474
	rs7596422	0.966	0.869	0.988	0.195	0.608
SCN2B	rs8192613	0.109	0.331	0.235	0.955	0.877
	rs8192614	0.964	0.358	0.115	0.275	0.923
SCN3B	rs3851100	0.279	0.804	0.672	0.956	0.578
	rs1783901	0.574	0.887	0.776	0.624	0.391
SCN8A	rs303777	0.374	0.682	0.989	0.617	0.794
	rs11169883	0.527	0.735	0.545	0.166	0.792
	rs303778	0.155	0.914	0.754	0.081	0.002

Linear regression using ANOVA test.

*p* value <.05 is significant.

**Table 5.** Regression analysis of SCN variants and epilepsy features.

Gene	SNP ID	gender	relapse	History of febrile seizure	periodic Seizure	Time to remission	Drug responsiveness	drug level	Family history of epilepsy	Epilepsy classification	Seizure classification
SCN1A	rs3812718	0.060	0.054	0.375	0.268	0.588	0.820	0.382	0.645	0.458	0.976
	rs2298771	0.398	0.118	0.781	0.268	0.632	0.914	0.987	0.552	0.220	0.685
	rs10167228	0.124	0.322	0.670	0.489	0.347	0.947	0.053	0.426	0.436	0.661
	rs6432858	0.430	0.104	0.797	0.281	0.585	0.882	0.975	0.609	0.216	0.695
	rs10194956	0.189	0.272	0.156	0.618	0.771	0.248	0.018	0.491	0.953	0.477
	rs2169312	0.084	0.156	0.006	0.660	0.383	0.098	0.310	0.067	0.669	0.859
	rs1461197	0.137	0.549	0.203	0.062	0.088	0.664	0.155	0.206	0.337	0.101
	rs13405797	0.105	0.257	0.023	0.734	0.772	0.068	0.289	0.035	0.635	0.731
	rs4667866	0.896	0.324	0.988	0.497	0.317	0.454	0.599	0.910	0.759	0.218
	rs1020853	0.098	0.523	0.667	0.197	0.704	0.347	0.718	0.431	0.185	0.980
	rs1381105	0.054	0.376	0.051	0.346	0.136	0.348	0.518	0.035	0.521	0.378
	rs2162600	0.088	0.436	0.026	0.720	0.501	0.116	0.308	0.065	0.656	0.841
	rs10182473	0.099	0.500	0.167	0.530	0.073	0.228	0.247	0.114	0.359	0.769
	rs16851381	0.896	0.324	0.988	0.497	0.317	0.454	0.599	0.910	0.759	0.218
	rs4667869	0.082	0.490	0.238	0.378	0.428	0.195	0.704	0.344	0.509	0.779
	rs10497275	0.896	0.324	0.988	0.497	0.317	0.454	0.599	0.910	0.759	0.218
	rs6432861	0.388	0.055	0.208	0.431	0.742	0.898	0.415	0.588	0.561	0.993
	rs7580482	0.173	0.587	0.484	0.905	0.445	0.970	0.053	0.466	0.430	0.693
	rs13383628	0.228	0.215	0.155	0.728	0.823	0.262	0.040	0.574	0.958	0.471
	rs6722462	0.256	0.585	0.191	0.068	0.075	0.807	0.147	0.466	0.325	0.096
	rs6432860	0.391	0.091	0.777	0.264	0.587	0.921	0.990	0.644	0.170	0.682
	rs994399	0.191	0.546	0.472	0.882	0.408	0.939	0.051	0.517	0.424	0.703
	rs1542484	0.382	0.062	0.591	0.268	0.713	0.671	0.628	0.586	0.457	0.974
	rs1461193	0.139	0.293	0.655	0.508	0.314	0.979	0.051	0.476	0.430	0.671
	rs11690959	0.212	0.197	0.148	0.654	0.781	0.284	0.039	0.541	0.948	0.482
	rs7601520	0.193	0.456	0.930	0.649	0.447	0.904	0.050	0.639	0.550	0.666
SCN2A	rs12467383	0.096	0.253	0.876	0.716	0.980	0.069	0.167	0.922	0.298	0.317
	rs1965757	0.022	0.690	0.072	0.733	0.347	0.334	0.670	0.762	0.708	0.090
	rs353139	0.099	0.104	0.040	0.814	0.787	0.047	0.395	0.368	0.680	0.419
	rs7581811	0.035	0.115	0.586	0.711	0.906	0.262	0.919	0.872	0.767	0.517
	rs2116658	0.642	0.542	0.500	0.542	0.939	0.553	0.690	0.267	0.502	0.165
	rs12993173	0.082	0.248	0.370	0.643	0.909	0.584	0.831	0.531	0.882	0.602
	rs4667485	0.167	0.875	0.609	0.011	0.815	1.00	0.695	0.319	0.555	0.994
	rs1469649	0.257	0.841	0.679	0.040	0.911	0.774	0.594	0.743	0.228	0.782
	rs1579865	0.407	0.559	0.492	0.769	0.989	0.300	0.452	0.097	0.547	0.131
	rs935403	0.197	0.666	0.131	0.337	0.461	0.271	0.946	0.897	0.853	0.014
	rs7592445	0.021	0.717	0.052	0.720	0.624	0.430	0.574	0.793	0.848	0.102
	rs2119067	0.540	0.367	0.050	0.753	0.712	0.349	0.515	0.023	0.062	0.975
	rs2119068	0.266	0.059	0.132	0.209	0.452	0.519	0.941	0.692	0.294	0.918
	rs10197716	0.489	0.656	0.226	0.055	0.928	0.986	0.536	0.070	0.070	0.422
	rs353112	0.851	0.980	0.271	0.006	0.279	0.354	0.203	0.769	0.851	0.209
	rs6740895	0.374	0.945	0.986	0.614	0.650	0.148	0.276	0.478	0.821	0.840
	rs3943809	0.044	0.426	0.828	0.716	0.984	0.067	0.325	0.898	0.297	0.320
	rs16850331	0.066	0.347	0.783	0.583	0.947	0.042	0.151	0.802	0.174	0.813
	rs12614399	0.049	0.452	0.485	0.580	0.980	0.034	0.181	0.641	0.170	0.844
	rs10182570	0.712	0.954	0.782	0.001	0.711	0.455	0.378	0.833	0.039	0.578
	rs1446579	0.427	0.518	0.268	0.310	0.334	0.260	0.808	0.757	0.525	0.371
SCN3A	rs7598098	0.024	0.277	0.980	0.350	0.751	0.972	0.571	0.194	0.923	0.235
	rs12615864	0.662	0.740	0.935	0.538	0.798	0.862	0.292	0.118	0.418	0.260
	rs16850186	0.636	0.382	0.489	0.716	0.847	0.535	0.706	0.848	0.165	0.154
	rs1439806	0.622	0.900	0.726	0.277	0.190	0.544	0.115	0.821	0.971	0.895
	rs7596422	0.090	0.567	0.088	0.602	0.564	0.169	0.011	0.474	0.651	0.753
SCN2B	rs8192613	0.798	0.125	0.730	0.465	0.367	0.963	0.464	0.135	0.655	0.864
	rs8192614	0.626	0.612	0.150	0.257	0.438	0.291	0.415	0.660	0.256	0.551
SCN3B	rs3851100	0.387	0.427	0.148	0.578	0.603	0.997	0.474	0.289	0.232	0.312
	rs1783901	0.246	0.754	0.999	0.114	0.489	0.173	0.929	0.578	0.141	0.073
SCN8A	rs303777	0.918	0.645	0.479	0.225	0.245	0.786	0.692	0.764	0.746	0.961
	rs11169883	0.393	0.806	0.989	0.623	0.111	0.781	0.019	0.105	0.249	0.058
	rs303778	0.381	0.906	0.316	0.019	0.434	0.572	0.705	0.556	0.178	0.329

Linear regression using Pearson's r test.

*p* value <.05 is significant.

According to the results displayed in Table 5, rs2169312, rs13405797, rs2162600 of SCN1A and rs353139 of SCN2A were in association with history of febrile seizure of epilepsy (*p* = 6e-3, .023, .026 and .040, respectively), whereas rs1965757, rs7581811, rs7592445 rs3943809 and rs12614399 of SCN2A and rs7598098 of SCN3A were correlated to gender of epileptic patients (*p* = .022, .035,.021,.044,.049 and .024, respectively). rs4667485, rs1469649, rs353112 and rs10182570 of SCN2A reported to be connected with periodic epilepsy in this study, *p* values = 0.011, 0.040, 6e-3 and 1e-3 respectively. rs353139, rs16850331, rs12614399 of SCN2A found to influence the drug responsiveness (*p* = .047, .042 and .034, respectively). Drug level was significantly associated with rs10194956, rs13383628, rs11690959 of SCN1A, rs7596422 of SCN3A and rs11169883 of SCN8A (*p* values = 0.018, 0.040, 0.039, 0.011 and 0.019 respectively).

The rs13405797 of SCN1A and rs2119067 of SCN3A were related to family history of epilepsy (*p* = 0.035 and 0.023). The rs10182570 of SCN2A was correlated to Epilepsy classification (*p* = 0.039), while rs935403 was of the same gene found in association with seizure classification (*p* = 0.014) (Table 5).

## Discussion

Epilepsy is a complex neurological condition that impacts the brain and cause seizure [24]. Genetic predisposition to epilepsy has been a fundamental part of the disorder aetiology [25]. Voltage-gated sodium channels are critical for genetics epilepsy, and these channels play a key role in mediating the electrical excitability. Thus, it is lucid that any genetic mutations in these gene coding channels can interfere the epilepsy development or progression. When the channels are activated by membrane depolarization, it will cause conformational change that increases the sodium ion influx in addition to cell depolarization and later the channels will be deactivated ending in resting of membrane potential [11]. This study investigated several genetic variants of SCN genes (SCN1A, SCN2A, SCN3A, SCN1B, SCN2B, SCN3B and SCN8A) and their association with epilepsy risk; these genes have been studied in this regard and conflicted results were reported [7]. rs3812718 is a common intronic variant that located in splice donor site, and it modifies alternative splicing of exon 5. We suggest that TT genotype of rs3812718 in SCN1A may be a protective factor against epilepsy and may decrease the risk of the disease in Saudi population. In contrast to our finding, rs3812718 was reported as a risk factor for GEFS + in Chinese population [26,27]. In one meta-analysis they revealed that the rs3812718 TT genotype was involved in high risk of developing drug resistance in epilepsy children [28].

We also discovered that in SCN1A gene, the variant genotypes of rs6432861 (CC) may influence the risk of epilepsy according to this study outcomes. We also propose that within the SCN2A gene, the C/CC of rs4667485 as well as the G/GG of rs1469649 may act as increased risk factor for epilepsy development and progression in Saudis. Moreover, The GATGCTCGGTTTCGCTACGCA haplotype of SCN2A gene was significantly related to epilepsy increased risk.

The other part of the current study explored the association between different clinical features of epilepsy and SCN genes variants. We found that these variants, rs1965757, rs7581811, rs7592445 rs3943809, rs12614399 of SCN2A and rs7598098 of SCN3A imply variation of epilepsy risk depending on the individuals’ gender. It has been indicated that men are more vulnerable to localization-related symptomatic epilepsies, whereas cryptogenic localization-related epilepsies were more frequent in women [29]. We also infer that SCN2A genetic variants, rs10194956 and rs4667869, may affect the duration of first seizure. Vitamin B12 deficiency may result in neurological problems (Benbir et al. 2006). In this regard, the concentration of vitamin B12 found to be associated with the rs303778 of SCN8A in epilepsy patients in this study. History of febrile seizure of epilepsy can be posed as an essential prognostic factor that can predict epilepsy development. We propose that the rs2169312, rs13405797, rs2162600 of SCN1A and rs353139 of SCN2A are risk factor for epilepsy among individual with history of febrile seizure. Furthermore, the rs13405797 of SCN1A and rs2119067 of SCN3A can be considered as epilepsy risk biomarkers within individuals have a family history of epilepsy.

Epileptic spasms are a type of seizure that last 1 to 2 s and spasms occur predominantly in infants. The prognosis of epileptic spasms is generally connected with deprived neurodevelopmental and behavioural outcomes [30]. In this study, we found that SCN2A gene variants (rs4667485, rs1469649, rs353112 and rs10182570) may be a risk factor for periodic epilepsy. Several Biomarkers of SCN1A gene have been proposed as risk factor to drug response and drug resistant of epilepsy. This prognosis clinical factor is important for clinical practice and medication enhancement [12]. In light of our finding, it seems more likely that the rs353139, rs16850331, rs12614399 of SCN2A found to influence the drug responsiveness to epilepsy. Otherwise, 31 have explored the clinical response of SCN1A variations to sodium channel blocking drugs in Chinese epilepsy patients and found no correlation. Another clinical parameter that was examined in this study is the drug level and was significantly associated with rs10194956, rs13383628, rs11690959 of SCN1A, rs7596422 of SCN3A and rs11169883 of SCN8A. We deduce that these variants may affect the demanded dose of AED for epilepsy. Classification of epilepsy and seizure types is significant for the primary diagnostic testing. We found that the rs10182570 of SCN2A was correlated to Epilepsy classification, whereas rs935403 of the same gene was found in association with Seizure classification.

In conclusion, within (SCN1A) gene the rs3812718, rs10194956, rs13383628, rs6432861 and rs1542484 may impact the risk of epilepsy. In addition, both rs4667485 and rs1469649 of SCN2A were significantly associated with epilepsy risk. For SCN3A, rs16850186 and the rs72550243 of SCN1B, we declare them as genetics factors that may influence the development and progression of epilepsy in Saudi population. Considering the clinical impact of epilepsy prognosis features, we propose voltage sodium channels as predictor factors for the clinical outcome of epilepsy. Molecular and Pharmacogenomics studies are highly recommended to elucidate the inter-individuals variability in response to AED in the light of different prognosis parameters.

The main limitations of this case-control study were gender and the relatively small sample number that could lead to selection bias. However other potential limitations were avoided such as bias was caused by population stratification, as the Saudi Arab population is a relatively homogenous population. Moreover, there were no significant differences between case and control groups in terms of basic demographic characteristics.

## Data Availability

The data used to support the findings of this study are available from the corresponding author upon reasonable request.
